# Prevalence of 3.7 and 4.2 deletions in Sudanese patients with red cells hypochromia and microcytosis

**DOI:** 10.1186/s13104-020-4933-5

**Published:** 2020-02-10

**Authors:** Hussam Ali Osman, Muzamil Mahdi Abdel Hamid, Rahimah Binti Ahmad, Mohamed Saleem, Sana Altahir Abdallah

**Affiliations:** 1 Department of Biotechnology, School of Pharmacy, Ahafad University for Women, Omdurman, Sudan; 2 Institute of Endemic Diseases, Medical Campus, University of Khartoum, Khartoum, Sudan; 3 Hematology Unit, Cancer Research Centre Institute for Medical Research Jalan Pahang, 50588 Kuala Lumpur, Malaysia; 4 Advanced Genomics SDN BHD (GenomixLAB), Kota Damansara, Malaysia; 5 Department of Pathology, Faculty of Medicine, Alneelain University, Khartoum, Sudan

**Keywords:** Alpha thalassemia, Multiplex Gap-PCR, Heterozygous/carriers, Deletion mutations

## Abstract

**Objective:**

Alpha-thalassemia is a genetic disorder characterized by deletions of one or more α globin genes that result in deficient of α globin chains reducing haemoglobin concentration. The study aimed to screen 97 patients with microcytosis and hypochromasia for the 3.7 and 4.2 alpha thalassemia deletion mutations.

**Results:**

Out of 97 patients screened, only 7 were carriers for the 3.7 deletion and all patients were negative for the 4.2 deletion. The 3.7 deletion was found in Foor, Hawsa and Rezagat Sudanese tribes. In the carriers of the 3.7 deletion, Red Blood Cells and Haematocrit were significantly increased. The Red Blood Cells were 7.23 ± 0.78 × 10^12^/L in adult males and 7.21 ± 0.67 × 10^12^/L in adult females while in children were 5.07 ± 0.87 × 10^12^/L. The mean cell volume and mean cell haemoglobin were significantly decreased, but the mean cell haemoglobin concentration slightly decreased. Haemoglobin levels didn’t revealed statistically significant decrease in adult males (11.7 ± 0.57 g/dL) and adult females (11.25 ± 0.64 g/dL), while in children were (11.6 ± 2.95 g/dL). Haemoglobin electrophoresis revealed two patients of the 3.7 and 4.2 negative were carriers for β-thalassemia. The study concluded that α^3.7^ deletion has frequency of 0.07 in Sudanese with hypochromasia and microcytosis.

## Introduction

Thalassemia is a hereditary blood disorder in which there is impaired synthesis of one or more globin chains of haemoglobin [1, 2]. Thalassemia syndromes can be conveniently classified according to the type of globin chain affected. Of these, there are two which are of special importance; they are called α-thalassemia and β-thalassemia. Others such as δβ, γδβ, δ, γ and εγδβ thalassemia are rare. Unlike β-thalassemia, the most common molecular defects in alpha thalassemia is due to large deletions involving either or both *HBA1* and *HBA2* genes. Most commonly recognised two α-globin gene deletions are --^SEA^, --^FIL^, --^MED^ and --^THAI^, while common single α-globin gene deletions are − α^3.7^ and − α^4.2^. Less frequently, α-thalassemia results from single-nucleotide variants. Historically, the distribution of α-thalassemia follows a pattern consistent with the degree of malaria endemicity and shows a highest prevalence in Africa with an allele frequency of 0.3–0.4 for − α^3.7^ deletion. Alpha thalassemia is also common in Southeast Asian countries like Thailand, Laos, and Vietnam [3, 4]. The α-thalassemia has a wide range of distribution globally, so annually 300,000 thalassemia patients are born. It affects 5–40% of the population in Africa and 40–80% in South Asia [3, 5]. The prevalence and frequency of the disease into an increase globally because of the migration worldwide [6].

Although the incidence and prevalence of alpha thalassemia is known to be increased in the African countries, its exact incidence or prevalence in Sudanese remains unknown, so we hypothesized there are many asymptomatic carriers for alpha thalassemia mutations masked in Sudanese community.

## Main text

### Methods

This is a cross sectional study, aimed to determine most common types of alpha thalassemia mutations in Africans (3.7 and 4.2 deletion mutations) on molecular basis and to correlate the finding with haematological parameters. Blood samples were analyzed at the Biotechnology Research Laboratory, Ahfad University and the department of Molecular Biology, Institute of Endemic Diseases, University of Khartoum. A total number of 97 patients with age ranged between 12 and 63 years old attended the clinical laboratories of six hospitals in Khartoum City (Khartoum Teaching Hospital, Omdurman Maternity Hospital, Gafer Ibn Oaf Paediatric Hospital, Mohamed Elamin Hamid Paediatric Hospital, Omdurman Teaching Hospital and Albuluk Paediatric Hospital) for routine check-up, were randomly selected on bases of the microcytosis (MCV < 80 fL in adults or < 75 fL in children), hypochromasia (MCH < 27 pg), normal ferritin level to exclude iron deficiency anaemia, no history and currently free of malaria infection and chronic disorders. Data were collected by observation of laboratory investigations and a well-designed questionnaire used for data collection. Complete blood counting was done using an automatic multi-parameter Haematology Analyzer (Sysmex kx-21) for in vitro diagnostic in the clinical laboratories in-order to detect the abnormalities. Alkaline haemoglobin electrophoresis method was carried out to detect the coinheritance of hemoglobinopathy mainly the sickle cell anaemia, heterozygous B-thalassemia, Hb H or Hb Barts, using SAS-MX Alkaline Hb-10 kit (Helena Bioscience Europe), then the types and percent of Hb were measured by densitometry method. Ferritin level was measured using full automated Cobas e411 (Roche, German) [7]. The genomic DNA was extracted using genomic DNA purification kit for whole blood (Jena Bioscience, Germany). The quantity and quality of extracted DNA was measured by a Nanodrope Spectrophotometer ND1000. Single tube multiplex Gap-PCR was done to screen all the 97 patients for the presence of the − α^3.7^ and − α^4.2^ deletions using the Platinum Multiplex PCR Master Mix (Invitrogen Applied Biosystem USA). The set of the primers used were supplied by Invitrogen Applied Biosystem, Germany). The sequence of the primers used were according to Chong [8]. A large (2.5 kilo base) region of the LIS1 gene (Lissencephaly 1gene) was co-amplified as an internal PCR control. Positive control genomic DNA samples were kindly provided by the Medical Research Institute—Kuala Lumpur, Malaysia. All data were analysed by SPSS version 14 and the *p*. values were calculated by paired sample t test.

#### Multiplex Gap-PCR

Before starting all reagents were allowed to thaw at room temperature, gently mixed by tube inverting and spin down. The reaction mixes were prepared according to the Platinum Multiplex PCR Master Mix manufacturer guidelines, [9] so each 25 uL reaction mix contained 12.5 uL Platinum Multiplex PCR Master Mix (2×), 5 uL GC enhancer (2×), 3.25 uL Primer Mix, 1 uL DNA (100 ng/uL) and 3.25 uL of Nuclease-free water. Senso Quest labcycler 48 (Germany) was used for PCR amplification with the initial denaturation at 95 °C for 5 min, followed by followed by 30 cycles of denaturation at 97 °C for 45 s, annealing at 60 °C for 1 min 15 s and 72 °C for 2 min 30 s, with a final extension at 72 °C for 5 min. PCR amplicons were analysed by electrophoresis on 1% agarose gel dissolved in 1 × Tris–Borate-EDTA buffer at 90 volts (current 40 amperes) for 2 h. The gels were stained and visualised under UV light.

### Results

**Fig. 1:**
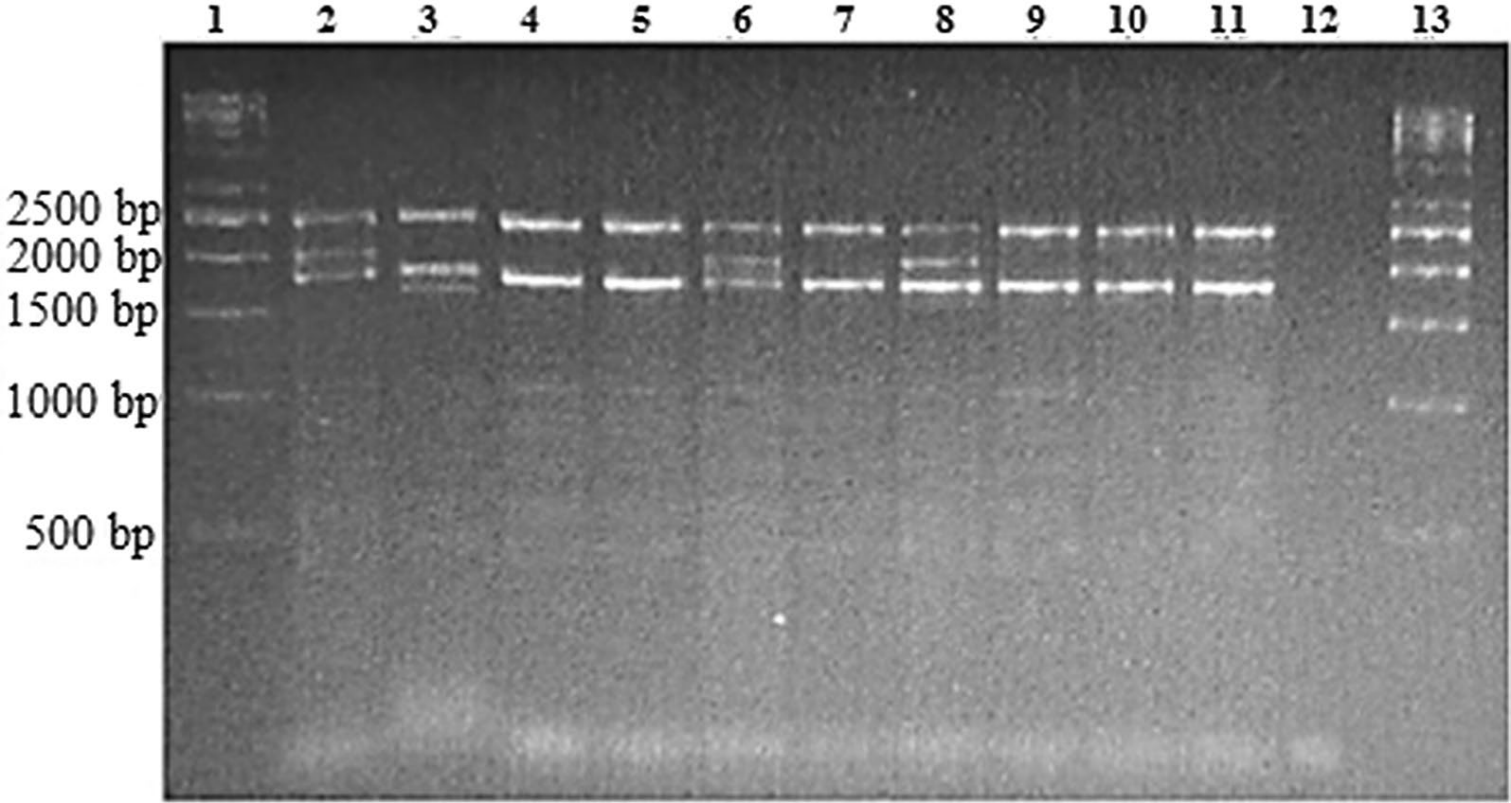
Multiplex PCR products on agarose gel electrophoresis. Lane 1 and 13 were the DNA ladder (1 Kb). Lane 2 and 3 were the 3.7 and 4.2 heterozygous control samples respectively. Lane 12 was the negative control. Lane 4, 5, 7, 9, 10 and 11 were examples of negative sample result. Lane 6 and 8 were example of heterozygous of 3.7 deletion mutation. LIS1 primer was used as internal control for the amplification of the LIS1 as a house keeping gene and the product band size was of 2.5 kb. The 3.7 and 4.2 deletions and the normal α_2_ product bands size were 2.1, 1.6 and 1.8 kb respectively

A total number of 97 patients were enrolled in the study, 67 of them were children (12–17 years old) including (30 males and 37 females) and 30 adults (18–63 years old) including (12 males and 18 females). All patients were Sudanese distributed in 36 Sudanese Tribes. On basis of the molecular biology investigations, 7 out of 97 individuals presented heterozygous − α^3.7^ deletion, while none was positive for − α^4.2^ deletion (Fig. 1) and these 7 carriers were belong to tribes of Foor, Hawsa and Rezagat that originated from West Africa.

The means of RBCs count for carriers were significantly increased in adult males and females (7.23 ± 0.78 × 10^12^/L and 7.21 ± 0.67 × 10^12^/L, respectively) while were normal (5.06 ± 0.87 × 10^12^/L) in children. Hb level was mildly decreased; which were 11.70 ± 0.57 g/dL, 11.25 ± 0.64 g/dL and 11.6 ± 2.95 g/dL in males, females and children, respectively. The means of haematocrit (HCT) were 38.70 ± 3.25%, 37.65 ± 2.33% and 35.06 ± 7.38% in males, females and children, respectively. MCV in adults was clearly decreased; 53.60 ± 1.27 fL, and 52.35 ± 1.63 fL in males and females, while in children was 69.20 ± 7.49 fL. MCH was 16.25 ± 0.92 pg, 15.60 ± 0.57 pg and 22.80 ± 3.44 pg in males, females and children, respectively. MCHC was 30.25 ± 1.06%, 29.90 ± 0.14% and 32.86 ± 1.64% in males, females and children, respectively. The means RDW_CV were 20.20 ± 1.70% and 21.05 ± 0.07% in the adults (males and females respectively) and in children were 14.86 ± 0.95%. The types of haemoglobin in the study population according to the electrophoresis result revealed, 2 beta thalassemia trait patients, have increased Hb A_2_ (˃ 3.5%), 11 patients have Hb AS and 77 patients have normal haemoglobin (Hb AA) and no other haemoglobin variants detected. All carriers have normal haemoglobin (Hb AA) and no Hb H or Hb Barts were found (Table 1).

**Table 1:** Haematological parameters of the study patients

Parameter	Group	3.7 and 4.2 −ve (No = 90)	3.7 heterozygous (No = 7)
Number distribution	Male	10	2
	Female	15	3
	Children	65 (28 ♂, 37 ♀)	2 (♂)
RBC (× 10^12^/L)	Male	5.55 ± 0.77	7.23 ± 0.78
	Female	4.61 ± 0.39	7.21 ± 0.67
	Children	4.98 ± 0.74	5.05 ± 0.87
Hb (g/L)	Male	14.62 ± 0.87	11.70 ± 0.57
	Female	12.00 ± 1.70	11.25 ± 0.64
	Children	10.79 ± 1.50	11.6 ± 2.95
PCV (L/L)	Male	43.36 ± 2.06	38.70 ± 3.25
	Female	36.60 ± 4.56	37.65 ± 2.33
	Children	33.00 ± 3.75	35.06 ± 7.38
MCV (fL)	Male	67.80 ± 7.64	53.60 ± 1.27
	Female	69.77 ± 8.05	52.35 ± 1.63
	Children	67.23 ± 4.97	69.20 ± 7.49
MCH (pg)	Male	23.44 ± 2.17	16.25 ± 0.92
	Female	23.18 ± 2.95	15.60 ± 0.57
	Children	21.99 ± 2.43	22.80 ± 3.44
MCHC (%)	Male	34.18 ± 0.93	30.25 ± 1.06
	Female	32.73 ± 1.79	29.90 ± 0.14
	Children	32.63 ± 1.70	32.86 ± 1.64
RDW-CV	Male	14.26 ± 1.98	20.20 ± 1.70
	Female	13.75 ± 1.26	21.05 ± 0.07
	Children	15.77 ± 2.36	14.86 ± 0.95
Frequencies of Hb electrophoreses result	AA	77	7
	AS	11	0
	↑A_2_	2	0
	↑F	0	0

The above table showed the Haematological parameters and Haemoglobin electrophoresis results in the study population. *RBC* red blood cells, *Hb* haemoglobin, *PCV* packet cell volume, *MCV* mean cell volume, *MCH* mean cell haemoglobin, *MCHC* mean cell haemoglobin concentration, *RDW-CV* red cells distribution width coefficient variation, *AA* adult haemoglobin, *AS* haemoglobin AS (carrier haemoglobin S), *A*_*2*_ haemoglobin A_2_, *F* foetal haemoglobin

### Discussion

Globally, the frequency of alpha-thalassemia is low, but in some tropical and subtropical areas the frequency of carriers could be high (80–90%). However, the disease is unexplored in Sudanese, mainly because of the limited accessibility to molecular diagnostic facilities in the country [10–13]. In this study we report that at least 7% of hypochromic microcytic anaemia patients were carriers of − α^3.7^ deletion. Since incidence and prevalence of this syndrome was not well described in local population, it is extremely likely that α-thalassaemia triat may have been confused with iron deficiency state, especially if it was not assessed [14–17].

The RBCs in adult carriers showed a significant increase in counting with significant association with the mutation “*P value *< *0.05*”, while in the children they were normal in count. The increase of RBCs count can be considered as a matter of compensation, a finding that have been reported before [18–20]. The haemoglobin concentration revealed mild anaemia in adults and children. This was previously reported [14], where alpha thalassemia trait patients were characterized by slight reduction in haemoglobin level. The MCV and MCH showed microcytosis and hypochromasia in adults, and this finding is consistent with many previous studies concerning the contribution of alpha thalassemia to microcytosis and hypochromia [20–27], while others [14, 28] reported slight microcytosis and hypochromasia or sometimes normal with alpha thalassemia trait. Unlike many other similar studies that have examined the red cell indices in alpha thalassaemia, present cohort showed significantly lower MCV (52–53 fL) giving an impression of additional pathology. The marked reduced MCV in the adult group, irrespective of their alpha thalassaemia carrier status, could be caused by the effect of coincidental chronic infection leading to anaemia of chronic disease.

The severe microcytosis and hypochromasia that is reported here might also be due to a coinheritance of the α^3.7^ allele with other deletion type or point mutation, such as AC deletion in vicinity of the initiation codon of the − α^3.7^ allele [29]. RDW_CV of the − α^3.7^ heterozygous group revealed anisopoikilocytosis, in adult males and females, while in children showed mild anisopoikilocytosis, this finding is consistent with previous studies [27, 30, 31].

The observed absence of − α^4.2^ deletion in this study does not completely rule out the presence of α+ thalassaemia in the general population as this study only focused on hypochromic microcytic anaemia patients. Since heterozygous α+ thalassaemia may have a completely normal blood count or trivial hypochromia and anaemia, more inclusive study is required to confirm the observation.

In conclusion we confirm the presence of α^3.7^ allele in Sudanese with marked microcytosis and hypochromasia, where the disease was unknown before. This suggests that, previous migration from West Africa crossing Sudan was the main cause of transmission of such type of mutation in Sudanese.

## Limitations

The negativity of type 4.2 deletion don’t exclude the presence of this type of mutation in Sudanese, so further studies should be done including large number of population from different sites to screen for phenotypes and genotypes types of alpha thalassemia among Sudanese in the future.

## Data Availability

All data generated or analyzed in this study are included in this manuscript.

## Abbreviations

CBC: Complete blood count
DNA: Deoxyribonucleic acid
FIL: Filipino
fl: Femto liter
g/dL: Gram per dice liter
g/l: Gram per liter
Hb: Haemoglobin
Hb A_2_: Hemoglobin A_2_
Hb F: Fetal haemoglobin
HbCS: Hemoglobin constant spring
HCT: Hematocrit
EDTA: Ethylenediaminetetra-acetic acid
Kp: Kilo base pare
LIS1: Lissencephaly 1
MCH: Mean cell haemoglobin
MCHC: Mean cell haemoglobin concentration
MCV: Mean cell volume
MED: Mediterranean
°C: Degree centigrade
PCR: Polymerase chain reaction
pg: Pico gram
RBCs: Red blood cells
RDW_CV: Red cell distribution width coefficient variation
SEA: Southeast Asia
SPSS: Statistical Package for Social Studies
STD: Standard deviation
THAI: Thailand
USA: United State of America
ul: Micro liter
−ve: Negative
WBCs: White blood cells
WHO: World health organization
α: Alpha
β: Beta
γ: Gamma
δ: Delta
ε: Epsilon
ζ: Zeta
